# Occurrence, Impact on Agriculture, Human Health, and Management Strategies of Zearalenone in Food and Feed: A Review

**DOI:** 10.3390/toxins13020092

**Published:** 2021-01-26

**Authors:** Dipendra Kumar Mahato, Sheetal Devi, Shikha Pandhi, Bharti Sharma, Kamlesh Kumar Maurya, Sadhna Mishra, Kajal Dhawan, Raman Selvakumar, Madhu Kamle, Awdhesh Kumar Mishra, Pradeep Kumar

**Affiliations:** 1 CASS Food Research Centre, School of Exercise and Nutrition Sciences, Deakin University, Burwood, VIC 3125, Australia; kumar.dipendra2@gmail.com; 2 National Institute of Food Technology Entrepreneurship and Management (NIFTEM), Sonipat, Haryana 131028, India; sheetaldeshwal1993@gmail.com; 3 Department of Dairy Science and Food Technology, Institute of Agricultural Sciences, Banaras Hindu University, Varanasi 221005, India; shikhapandhi94@gmail.com (S.P.); sbharti51997@gmail.com (B.S.); kamleshcfstbhu@gmail.com (K.K.M.); sadhnamishra2649@gmail.com (S.M.); 4 Department of Food Technology and Nutrition, School of Agriculture Lovely Professional University, Phagwara 144411, India; kajaldhawan42@gmail.com; 5 Centre for Protected Cultivation Technology, ICAR-Indian Agricultural Research Institute, Pusa Campus, New Delhi 110012, India; selvakumarsingai@gmail.com; 6 Applied Microbiology Lab., Department of Forestry, North Eastern Regional Institute of Science and Technology, Nirjuli 791109, India; madhu.kamle18@gmail.com; 7 Department of Biotechnology, Yeungnam University, Gyeongsan 38541, Gyeongbuk, Korea

**Keywords:** zearalenone, food and feed contamination, health issues, management strategies

## Abstract

Mycotoxins represent an assorted range of secondary fungal metabolites that extensively occur in numerous food and feed ingredients at any stage during pre- and post-harvest conditions. Zearalenone (ZEN), a mycotoxin categorized as a xenoestrogen poses structural similarity with natural estrogens that enables its binding to the estrogen receptors leading to hormonal misbalance and numerous reproductive diseases. ZEN is mainly found in crops belonging to temperate regions, primarily in maize and other cereal crops that form an important part of various food and feed. Because of the significant adverse effects of ZEN on both human and animal, there is an alarming need for effective detection, mitigation, and management strategies to assure food and feed safety and security. The present review tends to provide an updated overview of the different sources, occurrence and biosynthetic mechanisms of ZEN in various food and feed. It also provides insight to its harmful effects on human health and agriculture along with its effective detection, management, and control strategies.

## 1. Introduction

Extensive concerns have been raised over the years about the presence of fungal secondary metabolites in food and feed [1,2]. Amongst these secondary metabolites, mycotoxins are comprised of toxic metabolites of filamentous fungi that are chiefly produced by *Aspergillus, Fusarium,* and *Penicillium* species [3]. Mycotoxin contamination in food and feed cause acute and chronic mycotoxicosis, including teratogenic, carcinogenic, oestrogenic, neurotoxic, and immunosuppressive effects. Mycotoxins also serve as a crucial factor governing the safety for human consumption as they pose a serious threat to microbiological food safety and human health [3,4,5]. The incident of mycotoxin contamination may occur at any stages of culturing, harvesting and storage [6]. It is more protruding in areas with inefficient control over food quality, deprived production technologies, and poor storage surroundings that accelerate fungal growth and toxin production. Contamination of food and feed with mycotoxins has shown inevitable effects and raised widespread threats due to their less susceptible nature to any physical, chemical or thermal treatment [7].

Zearalenone (ZEN) is primarily produced by *Fusarium graminearum* and *Fusarium culmorum* and predominantly occurs in maize and other grain crops [7,8]. These species of *Fusarium* are generally found on plants primarily grown in temperate regions and contaminate foods of both plant and animal origin [1]. ZEN represents xenoestrogens having a chemical structure analogous to natural estrogens that permits its binding with estrogenic receptor sites leading to amplified estrogenicity. Exposure to this contaminant is accompanied by reduced levels of progesterone and serum testosterone in the bloodstream resulting in infertility and reduced incidences of pregnancy in animals like cows, pigs and rats [9,10]. It has also been shown to exert immunotoxic effects at low concentration levels. ZEN toxicity brings about numerous changes in the target cells by altering various metabolic events such as cell proliferation and apoptosis [11]. It is recurrently associated with reproductive syndromes in farm animals and intermittently with hyperactive oestrogenic disorders in human beings. There are numerous attestations of the fact that ZEN and its metabolites exert oestrogenic effects in pigs, sheep, and cattle amongst which pigs are the most susceptible to ZEN toxicity [12,13]. ZEN has been categorized as a Group 3 carcinogen by the International Agency for Research on Cancer (IARC) due to its unclassifiable carcinogenicity to humans with inadequate evidence [10]. However, owing to its continual incidence and extensive damage to both human and animal health, there is a need to adopt effective management strategies to control ZEN toxicity [14].

Insight of various immunotoxic and genotoxic effects of ZEN and its derivatives on human and animal health, there is an alarming attention towards the development of efficient and effective mitigation strategies against ZEN contamination [15]. As good storage and transportation facilities of agricultural commodities are not adequate to entirely hinder the occurrence of mycotoxin contamination in the food and feed chain, it is essential to embrace decent detection and decontamination strategies to mitigate the health risk and monetary losses [16,17,18]. This review tends to upgrade the information on various sources, chemistry, biosynthesis and occurrence of ZEN in food and feed. In addition to this, its impact on agriculture and human health along with probable detection and management strategies to safeguard the safety of food and feed will also be addressed in brief.

## 2. Major Source of Zearalenone

Fungal contamination and the subsequent production of mycotoxins are intrinsic to several food and feed across the globe [17,19]. Maize and other cereal crops such as barley, oats, rice, sorghum, rye and wheat, forming a comparatively major fragment of animal feed are primarily more susceptible to ZEN contamination [20]. Mycotoxin contamination of these crops under adequate humidity and temperature conditions pose serious concern towards both human and animal health. Also, foods prepared using the contaminated plant and animal products such as milk and meat products pose the utmost threat of mycotoxin contamination [21,22]. ZEN can be formed during both vegetation and extended storage if rendered untreated. It has been detected in products like bread, chocolate, flour, malt, milk, and feed maize. Given its hasty biotransformation and excretion by animals, the ingestion of this toxin together with meat is not very substantial [1]. Grains and vegetable protein in the animal feed serve as an essential source of nutrient for fungal growth rendering animal feed safety at risk [13]. The formation of mycotoxins in feed generally occurs during the pre-harvest stage and under inappropriate storage conditions [23]. Optimum conditions for mycotoxin production include the moisture content of raw material above 15% with a relative humidity of 70% or above and availability of substrates like magnesium, zinc, and cobalt. Other factors such as pH, optimum temperature (20–30 °C) and availability of oxygen also affect fungal growth [1].

## 3. Chemistry and Biosynthesis of Zearalenone

Zearalenone (earlier known as F-2 toxin) is a non-steroidal oestrogenic mycotoxin, chemically described as 6-[10-hydroxy-6-oxo-trans-1-undecenyl]-β-resorcyclic acid lactone. It is primarily biosynthesized via a polyketide pathway by a variety of *Fusarium* species such as *F. graminearum, F. culmorum,* and *F. cerealis* [12]. ZEN is formed as a result of successive reactions catalyzed by numerous multi-enzyme protein complexes that comprise polyketide synthases (PKSs). There are 15 PKSs that have been revealed in *F. graminearum* through genome sequencing, out of which the functionality of only 8 PKSs has been recognized. Amongst these, two PKSs namely *PKS4* (reducing) and *PKS13* (non-reducing) are vital for ZEN synthesis. These fungal PKS genes usually exist as a cluster that encrypts transcription factors, metabolic enzymes and transporters [24]. Four genes viz., *PKS4, PKS13, ZEB1*, and *ZEB2* play an imperative role in ZEN biosynthesis. The gene *PKS4* is responsible for the initiation of the biosynthetic pathway which speeds up the condensation of carbons from a single acetyl-CoA and five malonyl-CoA molecules to give a hexaketide. Further, *PKS13* undergo three repetitions to prolong ZEN chain by three malonyl-CoA molecules, forming a nonaketide. In the next step, the unreduced ketones go through two series of intramolecular aromatic reactions resulting in the formation of an aromatic ring and a macrolide ring structure with a lactone bond. The final stage is catalyzed by *ZEB1* to transform ZEL to ZEN [25].

**Figure 1:**
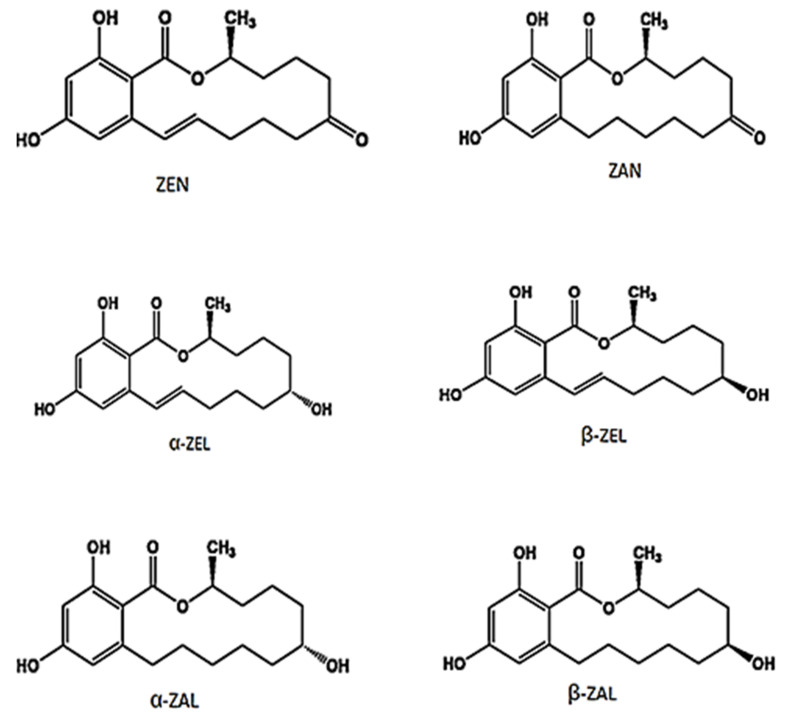
Chemical structure of zearalenone (ZEN) and its different metabolites.

The metabolism of ZEN is poorly understood in humans [26], however, studies in animal model suggest that ZEN is metabolized primarily to α-zearalenol (α-ZEL) and β-zearalenol (β-ZEL) [27,28] and the ratio of their concentrations is dependent on the type of animal species. It is further reported that α-ZEL and β-ZEL may reduce to α-zearalanol (α-ZAL) and β-zearalanol (β-ZAL) [29,30]. α-ZAL is metabolized predominantly into β-ZAL and, to a lesser extent, into zearalanone (ZAN) [31]. The chemical structure of ZEN and its different metabolites are presented in Figure 1.

ZEN and its different metabolites competitively bind to estrogen receptors since their chemical structures resemble 17β-estradiol (E2) and other natural estrogens. Shier et al. [32] determined the relative estrogenicity of ZEN and its different metabolites compared to E2 (92% for α-ZEL, 18% for α-ZAL, 3.5% for β-ZAL, 1% for ZEN, and 0.44% for β-ZEL) based on a proliferation assay on MCF7 human breast cells. Further, α-ZEL compounds are 2–4 times as estrogenic as ZEN and β-ZEL [33,34]. Moreover, α-ZEL was reported even to be 17 times as strong as α-ethynyl estradiol based on estrogen receptor gene activation bioassays [35] and the relative binding affinities to estrogen receptors was in decreasing order: α-ZEL, ZEN, and β-ZEL [36,37,38].

## 4. Genes Responsible for Zearalenone Production

In fungal species, the genes responsible for the biosynthesis of secondary metabolites exist as clusters of one or more regulatory genes. The majority of clusters involved in polyketide biosynthesis comprises of a single PKS gene and numerous gene encrypting enzymes [39]. ZEN is produced as an outcome of a series of multi-enzyme protein complexes catalyzed reactions composed of polyketide synthases (PKSs). These fungal PKSs are large multi-domain enzymes (type-I PKSs) with a repetitious function. Four adjacent genes viz., *PKS4*, *PKS13*, *ZEB1* and *ZEB2* as stated earlier are essential for the biosynthesis of ZEN biosynthesis and form a gene cluster [25]. Among these, a non-reducing *PKS13* is utilized during the biosynthesis due to the existence of ketone functional groups (as enol in resorcinol ring) in ZEN [40]. On the other hand, a reducing *PKS4* gene of *F. graminearum* is crucial for the formation of ZEN [41] as it catalyzes a vital step in ZEN biosynthetic pathway and also regulates the expression of other genes indulged in the process [42]. ZEN is primarily a polyketide that is solely synthesized from acetate-malonate fragments. The inhibitory effect on ZEN production can be exerted by diminishing the mycelia biomass and through down-regulation of genes *PKS4* and *PKS13* that are responsible for ZEN synthesis [3].

## 5. Occurrence in Food and Feed

ZEN synthesized by various *Fusarium* species like *F. cerealis, F. culmorum, F. crookwellense, F. equiseti, F. graminearum* and *F. semitectum* are common contaminants of various food and feed worldwide [43]. ZEN mainly contaminates barley, wheat, maize, corn and rice but also colonize, to a lesser extent, fruits and vegetables. Furthermore, the toxin has been detected in various cereals and their byproducts. ZEN derivatives (α-ZEL, β-ZEL, α-ZAL and β-ZAL) can also be detected in various food and feed infected with *Fusarium* in the field [44]. The predominant feature of ZEN distribution in cereal grains and animal feed is its occurrence with other *Fusarium* toxins including trichothecenes and fumonisins [45]. The occurrence of ZEN in various food and feed around the world is presented in Table 1.

## 6. Effects on Agricultural Food and Feed

ZEN is found to cause contamination at different stages of food chain leading to adverse health effects in both human and animal [46,47]. ZEN is commonly found in animal feeds and grains stored improperly [48] which makes them undesirable for consumption [49]. The European Commission has limited the ZEN content in feed materials to 2 mg·kg^−1^, except that in maize, the proposed maximum limit is 3 mg·kg^−1^ [50]. A study by Jia et al. [51] observed the effect of two *Fusarium* toxins, ZEN and deoxynivalenol (DON), by feeding piglets with contaminated feed. The results showed intestinal inflammation, change in the population of gut microbiota and reduced expression of protein namely claudin-4, even when fed with a low dose of ZEN and DON. The deleterious effect of ZEN on pig’s health such as inflammation and changes in the morphology of intestine even with low doses has also been confirmed by other several studies [52,53,54]. Different ZEN derivatives such as α-ZEL and β-ZEL are found in crops like rice, soybean and maize besides their contamination in processed food products like flour, beer etc. [12,33]. Moreover, as processing methods are unable to completely degrade the toxin, therefore, a tolerable daily intake for human adults of 0.25 µg/kg by weight has been recommended by the European Food Safety Authority for ZEN. Further to minimize the health risk, the European Union has specified limits for ZEN in food products. For instance, the maximum permissible limit of ZEN in unprocessed cereals is 100–200 µg/kg while for processed cereals, the limit has been reduced to 75 µg/kg [55].

**Table 1:** Occurrence of zearalenone in food and feed around the world.

Food/Feed Matrix	Country	Range (μg/kg)	Detection Technique	Reference
Food				
Wheat	China	10.1–3049	LC–MS/MS	[56]
Malt	Botswana	102–2213	TLC and HPLC	[57]
Amaranthus	Argentina	420–1980	TLC	[58]
Cheese snacks	Iran	1471	HPLC-FD	[59]
Wheat	China	5–1400	HPLC	[60]
Corn byproducts	Germany	369–1362	GC-MS	[61]
Wheat	Romania	327–1135	LC-MS/MS	[62]
Corn	Thailand	923	HPLC-GC-MS	[63]
Corn flour	Iran	889	HPLC-FD	[59]
Corn	Germany	48–860	GC-MS	[61]
Maize	Tanzania	729	UHPLC/TOFMS	[64]
Corn	Brazil	36.8–719	LC-MS/MS	[65]
Corn	Brazil	46.7–719	HPLC	[66]
Barley	Brazil	300–630	LC-MS/MS	[67]
Durum wheat	Tunisia	3–560	HPLC	[68]
Corn	Philippines	59–505	HPLC-GC-MS	[63]
Maize	Italy	453	HPLC	[69]
Sorghum	USA	443	GC-MS	[70]
Wort	Botswana	26–285 μg/L	TLC and HPLC	[57]
Wheat	Finland	1.9–234	LC-MS/MS	[71]
Wheat	Italy	7–231	HPLC-MS/MS	[72]
Maize	Iran	100–212	TLC	[73]
Soya meal	Germany	51–211	GC-MS	[61]
Beer	Botswana	20–201 μg/L	TLC and HPLC	[57]
Barley foods	South Korea	3.4–120	ELISA and LC	[74]
Wheat	Germany	17–104	LC-MS	[75]
Wheat	Hungary	50–98	ELISA	[76]
Wheat	Kenya	1–96	ELISA	[77]
Corn foods	South Korea	3.6–84	ELISA and LC	[74]
Maize	Argentina	0–83	TLC	[78]
Oat	Finland	76.9	LC-MS/MS	[71]
Wheat bran	Germany	3–67	HPLC-FD	[79]
Barley	Czech Republic	59.4	HPLC-MS	[80]
Corn	Brazil	55	TLC	[81]
Maize	Italy	53	HPLC	[82]
Rice	South Korea	21.7–47	RP-HPLC-FLD	[83]
Paddy rice	Turkey	42.9	HPLC-PDA/HPLC-FLD	[20]
Barley malt	German federal states	1.41–42.4	LC-MS/MS	[84]
Barley	Lithuania	10–41.4	LC-MS/MS	[85]
Corn flour	UK	6.5–40.8	HPLC-FD	[86]
Maize	Botswana	40	HPLC	[87]
Wheat	Brazil	40	TLC	[81]
Corn flour	Germany	2–40	HPLC-FD	[79]
Maize	Poland	18–39	HPLC	[88]
Wheat	Syria	4–34	HPLC-MS/MS	[72]
Barley	Croatia	32	ELISA	[89]
Sorghum	Ethiopia	19–32	HPLC	[90]
Maize	Turkey	28	HPLC-PDA/HPLC-FLD	[20]
Breakfast cereal	Spain	25	HPLC	[91]
Oat	Germany	21	GC-MS	[61]
Wheat, corn, oat bran, oat products	Germany	2–18	HPLC-FD	[79]
Banana	India	17	HPLC	[92]
Red pepper	Germany	2–17	HPLC-FD	[79]
Maize	Morocco	13.5–16.5	HPLC	[93]
Chilli oil/chilli powder/chilli sauce	UK	5–4/4.5–15.4/7.1	HPLC-FD	[86]
Wheat	Germany	15	GC-MS	[61]
Gluten-free food	Germany	2–14	HPLC-FD	[79]
Barley	Finland	13.7	LC-MS/MS	[71]
Corn	Indonesia	11–12	HPLC-GC-MS	[94]
Milk	Egypt	1.0–11.9	HPLC–FLD	[21]
Curry powder	UK	1.2–10.8	HPLC-FD	[86]
Semolina	Germany	2–9	HPLC-FD	[79]
Garlic pickle	UK	3–8	HPLC-FD	[86]
Fennel	UK	7	HPLC-FD	[86]
Bean	Germany	7	HPLC-FD	[79]
Corn	Argentina	3–7	HPLC	[95]
Corn flakes	Qatar	3.8–6.81	HPLC	[96]
Coriander	UK	3.6–6.7	HPLC-FD	[86]
Canned foods	UK	6.1	HPLC-FD	[86]
Hazelnut	Germany	6	HPLC-FD	[79]
Corn	South Korea	3.4–5.8	ELISA and LC	[74]
Wheat	Sweden	5	HPLC/ESI-MS/MS	[97]
Pumpkin kernel	Germany	4	HPLC-FD	[79]
Sunflower seed	Germany	2–4	HPLC-FD	[79]
Wheat germ/corn flakes	Germany	3/2–3	HPLC-FD	[79]
Wheat flour	Turkey	2.66	HPLC-PDA/HPLC-FLD	[20]
Wheat	Qatar	0.21–2.1	HPLC	[96]
Potato products	Germany	2	HPLC-FD	[79]
Chicken meat	Pakistan	0.85–1.83	HPLC	[22]
Rice	Qatar	0.18–1.4	HPLC	[96]
Wheat	Turkey	1.34	HPLC-PDA/HPLC-FLD	[20]
**Feed**				
Complete feed	China	10–3261.2	HPLC-FD	[98]
Cow feeding stuffs	Argentina	1200–3060	HPLC	[99]
Pig complete feed (powder)	China	10–835.4	HPLC	[100]
Corn and poultry feed	Indonesia	5.5–526	ELISA and HPLC	[101]
Duck complete feed	China	10–357.9	HPLC	[100]
Cattle, poultry and swine feed	Poland	0.07–349	HPLC-MS/MS	[102]
Pig complete feed (pellets)	China	10–329	HPLC	[100]
Broiler feeds	Thailand	2.22–263.51	HPLC	[103]
Complete feed for pigs	Norway	1.5–217.2	HPLC-FLD	[104]
Swine feed	Hungary	18–192	ELISA	[105]
Sheep compound feed	Spain	50–104.40	UPLC–MS/MS and UPLC–QTOF–MS	[106]
Cattle compound feed	Spain	88.2	LC-MS	[107]
Poultry feed mixture	Slovakia	3–86	RP-HPLC-FLD	[108]
Fish feed	Europe	67.9	HPLC	[109]
Chicken feed	China	61.59	UPLC-MS/MS	[110]
Swine compound feed	Spain	50	UPLC–MS/MS and UPLC–QTOF–MS	[106]
Chicken feed	Botswana	40	HPLC	[87]
Pig feed	South Korea	31.70	HPLC	[13]
Poultry feed	South Korea	0.24–26.80	HPLC	[13]
Pig feed/cattle feed/rabbit feed	China	18.78/14.43/10.46	UPLC-MS/MS	[110]
Starter feed	India	5.13–6.73	HPLC and TLC	[111]
Compound feeds	South Africa	0.56–1.85	ELISA	[112]

## 7. Effects of Environmental Factors on Zearalenone Production

The environmental conditions that affect the growth of *Fusarium* and thus the development of ZEN are precipitation, humidity, pH, temperature, composition of atmospheric gas and water activity (a_w_) [113]. a_w_ is the ratio of water vapour pressure in food to pure water vapour, at constant temperature and pressure [114]. It is a key factor in determining the microbial load in food. Lowering aw in foods reduces microbial growth and chemical reactions, thereby, enhancing the shelf-life of food products [115]. Hence, a_w_ is an indicator of water in food and its correlation with food quality, safety and stability [115].

*Fusarium* growth is promoted by relative humidity (90%), extended moisture period, moderate temperature (20–30 °C), degree of rainfall during pre-, post- and harvesting conditions as well as air currents [116]. The optimum temperature for *Fusarium* growth range between 25–30 °C and the optimum a_w_ between 0.980–0.995 [117]. Piacentini et al. [67] suggested weather to be an important factor since warm and humid conditions favor the *Fusarium* growth and ZEN production during the flowering period. Pleadin et al. [118] showed that the mold growth and ZEN production was further enhanced by the change in humidity from high to extreme during the growth and harvesting period. The chances of crop contamination with ZEN increase under a prolonged period of cool and wet weather conditions in temperate regions [68]. Habschied et al. [119] studied the effect of incubation time, temperature and a_w_ on ZEN concentration in crops. They found that at 20 °C, ZEN concentration in germ and bran increased with increase in incubation time whereas its concentration in flours increased with an increase in a_w_. Also, pH was observed to influence the ZEN production where alkaline pH favored the accumulation of the toxin at a lower incubation temperature (15 °C) [120]. The maximum production of ZEN was observed at pH 7 [121]. a_w_ is another factor affecting ZEN accumulation where the production of ZEN was observed to be higher at 0.995 a_w_ than at 0.950 a_w_, independent of temperature [122]. Further, Martins and Martins [123] observed that ZEN production was highest at 28 °C for 16 days followed by incubation at 12 °C (36.7 mg/kg) at the 35th day. Besides this, carbon dioxide level influenced the ZEN production by *F. graminearum* where maximum production occurred at 30 °C, 0.98 a_w_ and 400 ppm of CO_2_ [124].

## 8. Mechanism of Toxicity and Health Effects of Zearalenone

The contamination of crops with primarily *Fusarium* species leads to the production of ZEN that contaminates various food and feed [125]. Exposure to ZEN can display both acute and chronic effects. The chronic ingestion includes low-dose intake for a long time resulting in decreased productivity and resistance to pathogens and serves as a major concern relating to human and animal health [126,127]. The toxicokinetic study of ZEN deals with the rate at which it enters, gets absorbed and metabolized in the body and excreted out [10]. Various studies performed on animals have indicated rapid and wide absorption of ZEN (e.g., 80–85% in pigs) by the gastrointestinal tract (GIT) [128]. The GIT serves as a key site for primary interaction with the mycotoxin and is frequently exposed to the toxic agent [129]. In view of this, further extensive studies have been conducted to study the role of GIT in primary immune defense during the last decades [130]. On oral administration by human or animal, ZEN is promptly absorbed by the intestine and breaks down into α- and β-ZEL by α- and β-hydroxysteroid dehydrogenase, respectively. Thus, intestinal epithelial cells are exposed to toxic substances that bring out structural changes in intestinal villi and augment lipid oxidation process resulting in oxidative stress in the intestine [126,131,132,133]. Apart from this, ZEN has shown a significant genotoxic potential [134] and can persuade oxidative DNA injury [135]. ZEN can also lead to DNA fragmentation, cell cycle arrest [136], micronuclei formation and chromosomal aberrations [137]. Further, it exerts nephrotoxic and hepatotoxic effects [138] and encourages the development of hepatocarcinoma [139,140].

The most common toxic effect of ZEN is related to reproductive disorders with pigs being the most affected [141]. Pigs are mostly used as the model for research since they possess similar digestive and immune system as humans [142]. ZEN is similar to that of β-estradiol, therefore, it activates estrogen receptors [130]. Further, ZEN binds with estrogen receptors resulting in hormonal imbalance and can lead to reproductive diseases [143,144]. Studies have shown that ZEN can induce apoptosis of ovarian granulosa cells which are crucial for the follicular development and ovulation [145,146]. ZEN is involved in human hyperestrogenic syndromes besides causing reproductive disorders in farm animals [140]. Further, histological alterations in reproductive organ with decreased intracellular connections in testes was observed in the ZEN-treated mice [9]. In addition, exposure to ZEN during pregnancy and lactation can exert either reversible or irreversible effects on the offspring [130]. Because of potent estrogenic activity, ZEN has been convicted to chiefly affect reproductive functions in females [147]. Studies have indicated that exposure to estrogenic mycotoxin results in advanced puberty time [148]. It also interrupts with the estrous cycle and results in reproductive complications during fertilization, implantation and embryo development [149,150]. Moreover, a study by Massart et al. [151] indicated the contamination of breast milk with ZEN after consumption of contaminated food by the women. Studies conducted so far have revealed that the concentration of 1.0 ppm ZEN in the diet may exert hyper-estrogenic effects in pigs and a further increase in concentration may cause complications with conception leading to miscarriage and other numerous diseases [152]. Further, Mauro et al. [153] measured the serum ZEN metabolites in 48 overweight or obese women to study the association with the food intake. The results indicated that the level of ZEN was found in nearly all the surveyed woman, but its concentration varied with meat intake and the body mass index. Natural incidence of ZEN contaminated food has been identified as the cause of alterations in female reproductive organs and associated health problems [1,154].

Estrogens perform diverse biological functions such as female sexual differentiation and development, bone density maintenance and neuroprotective effects. These effects are the result of interaction between estrogen and estrogen receptor (ER), which triggers the target gene expression that encodes a protein of important biological function. They are generally converted to estrogenically inactive metabolites and eliminated from the body through urine and/or faeces [155]. Certain undesirable metabolites are produced as a result of hydroxylation and amination reactions during the biotransformation process. The enzyme CYP P450 is believed to be responsible for this [156,157,158]. This enzyme is most active in liver and intestine and is also responsible for the synthesis of fatty acids and steroids. The first stage in the metabolism of estrogens is the hydroxylation catalyzed by cytochrome P450 (CYP) enzyme. A chief metabolite of estradiol, 2-hydroxyestradiol, is primarily catalyzed by CYP1A2 and CYP3A4 in the liver, and by CYP1A1 in extrahepatic tissues. Though CYP1B1 mainly targets tissues of mammary, ovary and uterus, it explicitly catalyzes the 4-hydroxylation of estradiol. The 4-hydroxyestradiol further creates free radicals due to reductive-oxidative cycling with the corresponding semiquinone and quinone forms ultimately leading to cell damage. Modification in the expression level of estrogen metabolizing CYP isoforms not merely modifies the intensity of the estrogen action but may also modify the profile of its physiological effect in the liver and target tissues. In general, some CYP isoforms are produced by the substrates themselves resulting in improved and enhanced elimination from the body [155]. Further, the human CYP1B1 is regulated through estrogen receptor by estradiol and hence, suggest that the CYP enzymes involved in estrogen metabolism by estrogen itself would be responsible for the homeostasis of estrogens at local organs [155].

The study on interaction of ZEN with cellular component plays an imperative role in understanding the toxicokinetics of the toxin. Albumin is abundantly present in the plasma protein of the blood. Human serum albumin (HSA) sustains the oncotic pressure and the pH in the human circulation. It also plays an important function in the formation of various complex endogenous and exogenous compounds [125]. Thus, HSA shows a critical role in the pharmacokinetics and toxicokinetics of toxins, contaminants and drugs. In view of the important role of albumin in toxicokinetics of different toxins, interaction of ZEN with albumin has high biological importance. Various methods such as spectroscopy, ultrafiltration and molecular modelling have been employed to study the interaction. Fluorescence spectroscopy reveals that HSA forms a complex with ZEN indicating interaction with a binding constant of logK = 5.1 [125]. The strong interaction of ZEN-HSA complex suggests the potential biological importance and promotes precise understanding of toxicokinetic behavior of ZEN.

The functioning of ZEN as an immunomodulator and immunotoxic compound has also been explored. The immune response due to inflammation utilizes cytokines as a modulator. Cytokines play a key role in inflammatory responses and modulate humoral and cellular immune responses. Any imbalance in the cytokine production could intensify inflammatory reactions and cause pathological problems. Numerous studies have revealed a negative impact of ZEN on cells like white blood cells, B-cells, immunoglobins, T-cells and cytokines that are produced as a response to infection and perform immune functions [159,160]. Salah-Abbès et al. [159] reported that Balb/c mice treated with ZEN (40 mg/kg) for two weeks showed a significant decrease of total white blood cells, immunoglobulin levels (IgG and IgM), B-cells, T-cell subtypes (CD3þ, CD4þ, CD8þ), NK cells as well as pro-inflammatory cytokines. Another study by Islam et al. [140] evaluated how the immune system of mice gets affected when exposed to ZEN for two weeks. The results broadly showed the impact on the mice’s immune system with a decrease in CDC cells (CD4^+^, CD8^+^ and CD11c^+^) of the spleen and mesenteric lymph nodes. Antibodies’ levels were also examined in the serum to determine the humoral response and an increase in IgE while a decrease in IgM was observed. These observations indicate the immunotoxic nature of ZEN, however, more research is required to understand the underlying molecular mechanism of how ZEN differentially regulates immune cells and influences inflammatory responses against pathogens [140]. Further, mechanisms underlying many of these effects remain unrevealed to date.

## 9. Effects of Processing on Zearalenone

The occurrence of ZEN in agricultural products is known well. Owing to its harmful impact on health, it is important to opt for measures that can mitigate the effect. Thermal processing methods including boiling (100–125 °C), baking, frying and extrusion cooking (150 °C or above) reduce the ZEN, however, the reduction depends on temperature, pH and duration of processing [161]. Pleadin et al. [161] analyzed these thermal processing methods where the extrusion cooking was found to be a superior method to reduce ZEN contamination (up to 75%) followed by roasting which decreased the level up to 40%. In addition, extrusion cooking reduced ZEN by 83% in corn-based products [12]. Besides these, food irradiation has been investigated in corn kernel and flour where degradation of ZEN increased with increased doses of irradiation [162,163]. Gamma radiation resulted in a significant reduction in ZEN and the degradation increased with an increase in the moisture content of the product [164]. However, irradiations produce harmful residues. Therefore, a new cost-effective and environment-friendly technique like cold atmospheric pressure plasma (CAP) generating reactive oxygen and nitrogen species (RONS) in short operating time with an ability to react with a large number of mycotoxin molecules leaving no residues, has been developed [165]. Thus, different processing methods can contribute to lower down the toxicity level in food products.

## 10. Detection Techniques

The traditional analytical methods of detecting ZEN include chromatographic methods like HPLC [166,167], LC-MS/MS [168,169], and GC-MS [170]. In addition, a new method for purifying ZEN from rice culture of *Fusarium graminearum* using macroporous resin column coupled with high-speed counter-current chromatography was developed by Wang et al. [171]. These analytical methods have high sensitivity and specificity; however, the major drawback underlies with tedious sample preparation, long analysis time, high cost as well as unsuitable for on-site rapid inspection. To overcome these limitations, immunoassay methods were developed for simple, rapid as well as in-field monitoring of large-scale sample screening and detection with low cost and high sensitivity [172,173,174].

The most widely used immunoassay methods include enzyme-linked immunosorbent assay (ELISA) [175], rapid immunochromatographic assays (ICA) [176], immunochip [177], immunosensor [178], fluorescence polarization immunoassays [179,180], lateral flow immunoassay (LFA) [181], multiplex dipstick immunoassay [182], and suspension array [183]. Though these methods have high sensitivity and specificity, they require skilled manpower to operate. Therefore, Kolosova et al. [181] developed user-friendly membrane-based LFA requiring less staff training and analysis time. Later, LFA based on highly sensitive anti-ZEN monoclonal antibody was developed for rapid detection of ZEN in food and feed samples [184]. Recently, Jin et al. [185] have developed a novel dual near-infrared fluorescence-based LFA to determine ZEN in maize.

In addition, Li et al. [186] developed a 3D printed smartphone-based detection device integrated with solid phase latex microsphere immunochromatography platform (SIAP) for detecting ZEN in cereals and feed. It is also coupled with a user-friendly Android App which is self-written for analyzing, reporting, and sharing the results. The cut-off values of SIAP for ZEN in cereals and feed were 2.5 and 3.0 μg/kg, respectively, while the detection limits of the SIAP detection system for ZEN in cereals and feed were 0.08 and 0.18 μg/kg, respectively [186]. Further, Ren et al. [187] developed an anti-idiotypic nanobody-phage display-mediated immuno-polymerase chain reaction (PD-IPCR) method for detecting ZEN in cereals. The primers for PCR amplification were designed using specific DNA sequences encoding anti-idiotypic nanobodies and the detection limit for total ZEN in a cereal sample was observed to be 0.09 ng/mL [187]. Furthermore, biosensors-based methods have immense potential for detecting ZEN in food and feed. Caglayan and Üstündağ [188] developed an aptamer assay using attenuated internal reflection ellipsometry (AIR-SE) for detecting ZEN in cereals. The AIR-SE linked with the signal amplification through surface plasmon resonance has been observed to be a highly sensitive analytical tool in bio-sensing for the selective detection of ZEN in cereal-based products. The method showed better performance with the limit of detection (LOD) of 0.08 ng/mL and detection range between 0.01 and 1000 ng/mL [188].

## 11. Masked Mycotoxins as a Major Concern in Detection

As per Rychlik et al. [189], the “matrix-associated” mycotoxins form covalent bonds, complexes and/or are dissolved or trapped in the matrix while the “modified mycotoxins” include both “biologically and chemically modified” mycotoxins. Further, the term “masked mycotoxins” is referred to as “biologically modified” mycotoxins conjugated by plants [189]. These “modified” forms remain undetected by the routine analysis techniques [190]. The most plentiful derivatives of ZEN include α-ZEL and β-ZEL. Three phases of chemical modifications of ZEN have been observed during the plant metabolism. Phase I involves reduction, oxidation, or acetylation of the parent mycotoxin into a derived molecule of a higher toxicity level (e.g., α-ZEL). Phase II consists of the enzymatic transformation of the reactive groups through conjugation such as glucosidation and sulfation to form more hydrophilic compounds which can facilitate the elimination of the masked mycotoxins [169,191,192]. Phase III consists of compartmentalization of mycotoxins into the vacuole of plant or binding to the cell wall [12,193,194,195].

ZEN is efficiently transformed to its glucose conjugate after its production by several *Fusarium* species during infection of cereals and maize [196]. ZEN is reduced to α-ZEL and β-ZEL and then produces glucose conjugates of the respective compounds, especially Z14G. Further, ZEN and its metabolites have been observed to be transformed into conjugated compounds like glucosides, malonylglucosides, dihexosides, and pentosylhexosides by *Arabidopsis thaliana* [197]. A study by Schneweis et al. [75] on ten wheat grain samples showed the relative proportion of Z14G to ZEN to be around 27%. Contrary to this, no traces of Z14G, α- or β-ZEL, α-zearalenol-14-β-d-glucopyranoside (α-ZELG) or β-zearalenol-14-β-dglucopyranoside (β-ZELG) were reported in 84 cereal-based products analysed by Vendl et al. [198]. However, zearalenone-14-sulfate (Z14S) was reported in different wheat-based products like flour, bread, biscuits, wheat flakes, bran flakes, muesli, crackers, and snack bars with the highest quantity being 6.1 µg/kg in bran flakes [198]. Huang et al. [199] developed a sensitive and rapid method of ultra-high performance liquid chromatography combined with electrospray ionization triple quadrupole tandem mass spectrometry (UHPLC–ESI–MS/MS) for the simultaneous determination of aflatoxin M1, ochratoxin A, ZEN and α-ZEL in milk. Similarly, Han et al. [200] and Belhassen et al. [201] developed a rapid and sensitive UHPLC–MS/MS method to simultaneously determine total ZEN (free + conjugated) and its five metabolites (α-ZEL, β-ZEL, α-ZAL, β-ZAL, and ZAN) content in traditional Chinese medicines and human urine samples, respectively.

Further, ZEN conjugates across different matrices are enlisted in Table 2. Mycotoxins, occurring in conjugated form, i.e., either in soluble or incorporated into/associated with/attached to macromolecules, can transform back into their parent forms during metabolization by living plants, fungi, and mammals or after food processing. Thereby, pose a serious concern for human and animal health [193,202,203,204,205,206,207]. Further, these transformations can be achieved by a hydrolytic process involving either alkaline, acidic or enzymatic methods [208,209]. Hence, these hydrolytic methods along with in vitro digestion followed by detection with chromatographic techniques like LC/MS/MS and confirmation by methods like ELISA can be applied for modified ZEN to ensure the safety of food and feed.

**Table 2:** Zearalenone conjugates across different matrices.

Origin	Conjugated Zearalenone	Matrix	Reference
Fungal conjugates	Zearalenone 4-sulfate	*Fusarium graminearum, Rhizopus arrhizus*	[191,210]
	Zearalenone 4-glucoside	*Rhizopus* sp.	[211]
Plant conjugates	α-/β-Zearalenol 4-glucoside	Maize cells	[212]
	Zearalenone 4-glucoside	Maize cells, wheat	[75,196]
	Zearalenone dihexoside, α-Zearalenol dihexoside, β-Zearalenol dihexoside, Zearalenone malonylhexoside, α-Zearalenol malonylhexoside, β-Zearalenol malonylhexoside, Zearalenone pentosylhexoside, α-Zearalenol pentosylhexoside, β-Zearalenol pentosylhexoside	*Arabidopsis thaliana*	[197]
	Palmitoyl zearalenone	*Fusarium*-infected banana	[92]
Mammalian conjugates	Zearalenone 3-glucuronide	Urine	[213]
	Zearalenone 4-sulfate	Urine	[214,215]

## 12. Degradation Kinetics

The degradation methods have been categorized into physical, chemical, enzymatic and biological methods. Physical methods involve washing, sorting, grinding, hulling, adsorption, thermal treatment, and application of UV and gamma radiations [216]. The degradation of mycotoxin on increasing temperature and pH follows first-order reaction indicating greater destruction at high temperature and pH [217]. Further, adsorption methods adsorb mycotoxins directly and neutralize them, thus, making the mycotoxins ineffective [1]. Graphene oxide (GO) with amphiphilic didodecyl dimethyl ammonium bromide (DDAB) molecules has effectively adsorbed ZEN from maize oils [218]. Other adsorbents include organo-montmorillonites (OMts) [219], modified montmorillonites [220], montmorillonite clay with *Cymbopogon citratus* [221], organo-rectorites modified with different quaternary ammonium salts [222], organozeolites [223], talc and diatomaceous earth [224], activated carbon [225] and kaolin modified with octadecydimethilbenzyl ammonium [226].

Chemical methods are based on the application of chemicals such as ammonia, hydrogen peroxide, sodium hypochlorite and ozone [1]. Since ozone is safe, efficient and environmental friendly, it is widely used for detoxification of the toxin [227]. A study by Qi, et al. [228] indicated that ozone has the potential to significantly reduce ZEN in naturally contaminated corn without affecting its quality. Ozone reduces the toxicity of ZEN by forming degradation ozonolytic products which are less toxic than the original mycotoxin [229]. The reaction rate constant and degradation rate of ZEN increased with ozone concentration and treatment time [228] and this ZEN degradation by ozone followed first-order kinetic [230]. A study by Rogowska et al. [1] has suggested 83.9% ZEN degradation with the use of 10% hydrogen peroxide at 30 °C for 16 h. Su et al. [23] have also suggested the potential ability of vitamin C to reduce the toxicity of ZEN in animal models.

Both physical and chemical methods result in loss of nutrients and are regarded as expensive and ineffective, so biological strategies have been developed [216]. Biodegradation methods involve the use of microorganisms with high degradation efficiency, minimal environmental hazards with a lower reduction in nutritional and textural food quality [4]. The microorganisms degrade mycotoxin via two pathways: first through special structures in the cell wall which absorb the ZEN, reduce their exposure and thus achieve detoxification. Second through biotransformation of ZEN into less toxic compounds by metabolizing them [231]. Chlebicz and Śliżewska [232] have shown the detoxification properties of *Lactobacillus* and *S. cerevisiae* strains towards ZEN where the toxin decreases within 6 hours of incubation. *S. cerevisiae* degraded ZEN through intra- and extra-cellular production of enzyme which acted against oxidative stress and cell death and also assisted cell wall adsorption of toxin [216]. Further, *Lactobacillus pentosus* strains effectively reduced ZEN by binding 30–83% of ZEN and the binding capacity increased with increased ZEN concentration as determined by Freundlich isotherm [233]. In addition, *Lactobacillus plantarum* reduced ZEN by binding them into pellets [234]. The proteins in the extracellular extracts of *Acinetobacter* sp. SM04 and *Bacillus natto* CICC 24640 bring about the biodegradation of ZEN [235,236]. *Streptomyces rimosus* (K145, K189) reduces ZEN by producing toxin degrading enzymes [237]. Also, *Bacillus* strains RC1A, RC3A and RC6A degraded ZEN by extracellular metabolite, AHL enzyme [18]. Besides this, the combination of *B. subtilis* SP1, *B. subtilis* SP2, *C. utilis* and extracts of *A. oryzae* helps in biodegradation of ZEN [238]. *Bacillus amyloliquefaciens* ZDS-1, *Bacillus subtilis*, *Bacillus velezensis* Strain ANSB01E, *Bacillus amyloliquefaciens* H6, *Aspergillus niger* strain FS10, *Lysinibacillus* sp. ZJ-2016-1, *Pseudomonas alcaliphila* TH-C1, *Pseudomonas plecoglossicida* TH-L1, and *Rhodococcus pyridinivorans* K408 Strain also showed biodegradation of ZEN with a significant reduction in the mycotoxin level in food products either by adsorption of the toxin or through enzyme production which degraded the toxin [239,240,241,242,243,244]. Biodegradation using enzymes utilizes lactonohydrolase annotated as Zhd518 from *E. coli* and *P. pastoris* GSZ; lactonase from *Gliocladium roseum*, named ZENG; zearalenone lactonohydrolase (ZHD101) from *Clonostachys rosea*; laccase from *Trametes versicolor*; peroxidase (POD) from soya bean and rice bran; and a recombinant fusion enzyme (ZHDCP), a combination of hydrolase (ZHD) and carboxypeptidase (CP) having the potential to degrade the ZEN, hence, termed as ZEN-degrading enzymes [207,245,246,247,248].

## 13. Management and Control Strategies

Infection of crops by *Fusarium* growth and accumulation of ZEN has been a major concern for food and feed quality and safety, leading to economic losses. Hence, interventions to reduce contamination should be designed [249]. These interventions involve good agricultural practices (GAP’s) and good manufacturing practices (GMP’s) [250].

Pre-harvest strategies include weed eradication, soil analysis, application of herbicide, fungicide, and insecticide for pest and fungal eradication, seedbed treatment, decontamination of seeds, crop rotation, tillage and ploughing, fertilizers for nutrient enrichment, and genetically modified plants for mycotoxin suppression [50,251,252]. Tillage and ploughing in soil cultivation bring the contaminated part into the upper soil and can be removed so that the next crop is not infected [253]. Use of fertilizers especially nitrogen increases the growth of *Fusarium* along with the positive effect on plant growth and thus the contamination by mycotoxin increases [254]. Crop rotation helps in limiting the recontamination of crops in which *Fusarium* prone crops are cultivated with crops not prone to contamination [255]. Further, the strategies involving the cultivation of resistant varieties, excessive use of chemicals such as pesticides and insecticides, and extensive crop rotation programs have adverse effects on both human and environment. Therefore, novel strategies such as the use of biological control agents (BCA’s) which suppress the growth and colonization of harmful pathogens have been developed recently [256]. Abdallah et al. [257] have demonstrated that *Epicoccum* and *Sordaria*, endophytic fungi controlled the growth of *Fusarium graminearum* and hence, the production of ZEN in maize.

Post-harvest strategies include minimizing the time between harvesting and drying, efficient drying to a moisture content less than 14%, effective cleaning of maize before storage, hygiene and management of storage containers, clear specifications and traceability from field to store, absence of pests in storage areas, appropriate storage conditions in terms of temperature and moisture control and modified atmosphere storage using sulphur dioxide which is harmful to pathogenic microorganisms [258]. Other post-harvest strategies include physical, chemical, and biological decontamination of mycotoxin in agricultural products [250]. In the coming years, the foundation of such pre- and post-harvest strategies should be strengthened to prevent the mycotoxins from entering the human and animal food chain.

## 14. Conclusions

The pervasive contamination of various food and feed with mycotoxins poses serious threats to human and animal wellbeing as well as commercial trade across the globe. The instance of contamination can occur at any stage from pre- to post-harvest periods due to improper handling, storage, and distribution facilities. Insight of various deleterious effects of mycotoxin consumption such as teratogenic, carcinogenic, oestrogenic, and immunosuppressive effects, there is a need to develop efficient technologies for detection, decontamination, and management of these mycotoxins to prevent a threat to both human and animal with assured safety and security of food and feed. ZEN, an estrogenic mycotoxin, can contribute extensively to various hormone-dependent diseases. The development of effective neutralization and decontamination strategies for ZEN stands obligatory to mitigate its adverse effects as this toxin is highly resistant to various processing conditions. A comprehensive understanding of the biosynthetic mechanism stands essential for the development of effective control strategies. Various analytical methods such as HPLC, LC-MS/MS, and GC-MS have been developed for the detection of ZEN with different detection limits. Despite these, various other electrochemical, colorimetric, fluorometric, refractometric could be utilized for ZEN detection in food and feed. Furthermore, numerous physical, chemical and enzymatic methods could be used for decontamination of ZEN in food and feed for their safe consumption. Further advancement in these technologies and the adoption of novel detection and decontamination techniques is the need of the hour to embark the vision of attaining food and feed safety and security across the globe.

## Data Availability

Not applicable.
